# A rare case of angioleiomyoma of the knee: a case report

**DOI:** 10.4076/1757-1626-2-7885

**Published:** 2009-08-25

**Authors:** Talal Al-Jabri, Sunil Garg, Sudhir Rao

**Affiliations:** 1 Queen Mary’s HospitalFrognal Avenue, Sidcup, DA146LTUK; 2 Trauma and Orthopaedics, Queen Mary’s HospitalFrognal Avenue, Sidcup, DA146LTUK

## Abstract

We report a case of an angioleiomyoma occurring in a 40-year-old Kenyan female. The patient presented with recurrent pain and a soft tissue swelling at the posteromedial aspect of her right knee. Angioleiomyomas are benign soft tissue tumours, which occur most commonly in the skin of the lower extremities in middle-aged females. They very rarely occur in the knee and are treated curatively with resection. A brief literature review is included.

## Introduction

Angioleiomyomas, or vascular leiomyomas, are infrequent benign tumours originating from smooth muscle cells of arterial or venous walls. They were first described in 1937 by Stout AP [1], and since then several authors have published successive opinions regarding their origins. Some suggested that these tumours might be vascular malformations whilst others suggested that they could be a specific type of hamartoma [2]. Since their first description, angioleiomyomas have been classified into solid, cavernous and venous histological types [3]. They commonly affect the lower extremities however rarely affect the knee. We report a case of an angioleiomyoma of the knee. To the best of our knowledge only a minority of individual cases have been reported [4-8].

## Case presentation

A healthy 40-year-old Kenyan lady was referred to our hospital with an 8-year history of recurrent pain at the posteromedial aspect of her right knee. The onset of pain was sporadic and it had become progressively worse over the last few months thus reducing her mobility. Cold exposure and light touch often exacerbated the pain and conservative therapy with analgesia and local bandaging was of no avail. She underwent steroid injections in Germany at the onset of her symptoms 8 years ago, however this provided little relief.

**Figure 1.**
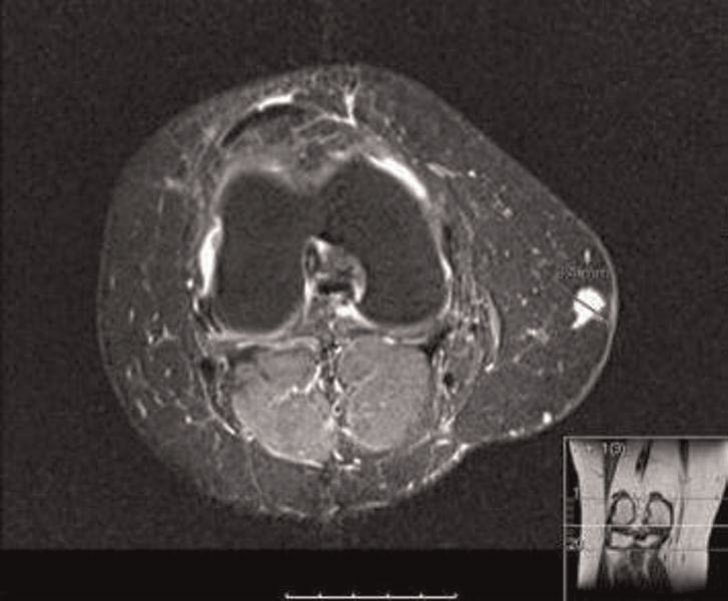
MRI scan showing angioleiomyoma in the right knee.

On physical examination the tumour presented as a subcutaneous swelling at the posteromedial aspect of her right knee. This was tender to palpation and owing to its location was initially thought to be a medial cutaneous neuroma. An MRI scan was also suggestive of this diagnosis (Figure 1).

Definitive treatment involved excision of the tumour via a medial incision to the right knee. The lesion was easily visualized and was attached to a very small cutaneous nerve found running in a transverse fashion across it. This small cutaneous nerve was sacrificed under traction in order to free the lesion which was then completely excised.

**Figures 2. (A and B):**
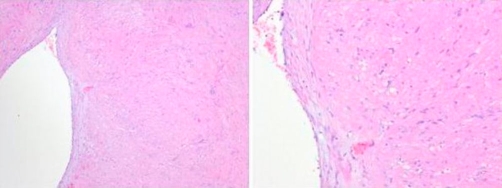
Microscopic images of the angioleiomyoma showing smooth muscle bundles and a vessel.

Macroscopically, the lesion was a firm white tumour measuring 10 mm × 8 mm × 6 mm. The histological appearance was consistent with that of an angioleiomyoma (Figures 2A and 2B). The patient experienced a complete resolution of her symptoms postoperatively.

## Discussion

Angioleiomyomas are benign soft tissue tumours composed of smooth muscle cells derived from vascular walls. They are more common in the lower extremities than in the upper extremities [2,9]. In addition, one study found that women developed lower extremity angioleiomyomas twice as commonly as men, and men developed upper extremity tumours more commonly than women [3,9]. The more painful solid tumours occurred predominantly in the lower extremities in females whereas cavernous tumours were found more frequently in upper extremity tumours [9]. It is uncertain why the distribution of angioleiomyomas follows this pattern however, it has been suggested that certain mechanical factors and tissue oestrogen levels may be implicated [2].

Pain, tenderness and temperature sensitivity are common findings [6]. It is unclear as to why or how cold exposure worsens pain, however Morimoto [3] proposed that pain in solid angioleiomyomas occurred following a period of contraction of vessels in the tumour which may cause ischaemia.

Most angioleiomyomas have a duration of between 4 to 7 years depending on tumour type [9]. Our patient had the tumour for approximately 8 years, which is longer than most previous cases. Reportedly most angioleiomyomas occur in the 4^th^, 5^th^ and 6^th^ decades of life and more than 80% of angioleiomyomas are less than 2 cm in diameter [3,9]. These findings are consistent with our patient.

Three case reports in the literature [4-6] briefly described that steroid injections and bandaging were of little use [4-6]. Our patient also stated that these did not alleviate symptoms.

MRI is useful in the workup as it delineates the extent of the tumour making excision easier [6]. Okahashi et al. [6] also used the ischaemic test described by Hildreth in 1970 for glomus tumours. It was found to be positive in their patient suffering from an angioleiomyoma in the knee [6,10]. This test tests for a vascular component to a tumour by abolishing pain after inflating a cuff on the effected extremity to above systolic pressure [10]. There may be a place for this diagnostic tool in clinical practice.

Angioleiomyomas should be considered in the differential diagnosis of a soft tissue mass as this will expedite treatment. Excision to date is curative in all patients in the literature and recurrence is very rare however if a tumour does recur one must consider leiomyosarcoma as a diagnosis [5,11].
